# Increased Bacterial Load and Expression of Antimicrobial Peptides in Skin of Barrier-Deficient Mice with Reduced Cancer Susceptibility

**DOI:** 10.1038/JID.2015.383

**Published:** 2016-01

**Authors:** Ken Natsuga, Sara Cipolat, Fiona M. Watt

**Affiliations:** 1 Cancer Research UK Cambridge Research Institute, Li Ka Shing Centre, Cambridge, United Kingdom; 2 Department of Dermatology, Hokkaido University Graduate School of Medicine, Sapporo, Japan; 3 Centre for Stem Cells and Regenerative Medicine, King’s College London, London, United Kingdom

## Abstract

Mice lacking three epidermal barrier proteins—envoplakin, periplakin, and involucrin (EPI-/- mice)—have a defective cornified layer, reduced epidermal γδ T cells, and increased dermal CD4^+^ T cells. They are also resistant to developing skin tumors. The tumor-protective mechanism involves signaling between Rae-1 expressing keratinocytes and the natural killer group 2D receptor on immune cells, which also plays a role in host defenses against infection. Given the emerging link between bacteria and cancer, we investigated whether EPI-/- mice have an altered skin microbiota. The bacterial phyla were similar in wild-type and EPI-/- skin. However, bacteria were threefold more abundant in EPI-/- skin and penetrated deeper into the epidermis. The major epithelial defense mechanism against bacteria is production of antimicrobial proteins (AMPs). EPI-/- skin exhibited enhanced expression of antimicrobial peptides. However, reducing the bacterial load by antibiotic treatment or breeding mice under specific pathogen-free conditions did not reduce AMP expression or alleviate the abnormalities in T-cell populations. We conclude that the atopic characteristics of EPI-/- skin are a consequence of the defective barrier rather than a response to the increased bacterial load. It is therefore unlikely that the increase in skin microbiota contributes directly to the observed cancer resistance.

## Introduction

In cells of the outermost epidermal layers, the plasma membrane is replaced by a cornified envelope, formed by transglutaminase-1-mediated crosslinking of proteins and lipids (Natsuga, 2014). This insoluble structure is a key component of the epidermal barrier that protects the body from environmental assaults (Candi et al., 2005). Defects in the epidermal barrier are linked to atopic dermatitis in humans and mice (Natsuga, 2014). Mice triply deficient in three cornified envelope precursors—*envoplakin*, *periplakin*, and *involucrin* (EPI-/- mice)—have epidermal barrier defects, a reduction in epidermal γδ T cells, and an increase in dermal CD4^+^ T cells (Sevilla et al., 2007).

EPI-/- mice are highly resistant to developing skin tumors when treated with chemical carcinogens (Cipolat et al., 2014). This is due, at least in part, to an exaggerated atopic response to the tumor promoter 12-O-tetradecanoylphorbol-13-acetate (TPA), including upregulation of the keratinocyte stress-associated antigen Rae-1, which binds natural killer group 2D (NKG2D) on immune cells. NKG2D ligands are currently being evaluated as therapeutic targets in a variety of cancer types (Spear et al., 2013).

Rae-1–NKG2D interactions are not only implicated in cancer (Cipolat et al., 2014, Strid et al., 2008) but also mediate host responses to bacterial and viral infection (Hamerman et al., 2004, Lodoen et al., 2004, Strid et al., 2008, Strid et al., 2009). Given the strong evidence linking the gut microbiota, chronic inflammation, and cancer (Shanahan and O'Toole, 2014), we speculated that the skin microbiome might be altered in EPI-/- mice. Human skin is covered by wide variety of commensal microbiota, which vary in composition according to body site, age, and health status (Grice and Segre, 2011). In addition, studies suggest a regulatory role for skin commensal bacteria in skin infection and inflammation (Lai et al., 2009, Naik et al., 2012). We therefore investigated whether the skin microbiome differs between EPI-/- and wild-type (WT) mice and whether it influences keratinocyte expression of Rae-1 and antimicrobial proteins (AMPs), natural antibiotics that are effectors of the innate immune system (Gallo and Hooper, 2012).

## Results

### Altered abundance and distribution of bacteria in the skin of EPI-/- mice

**Figure 1:**
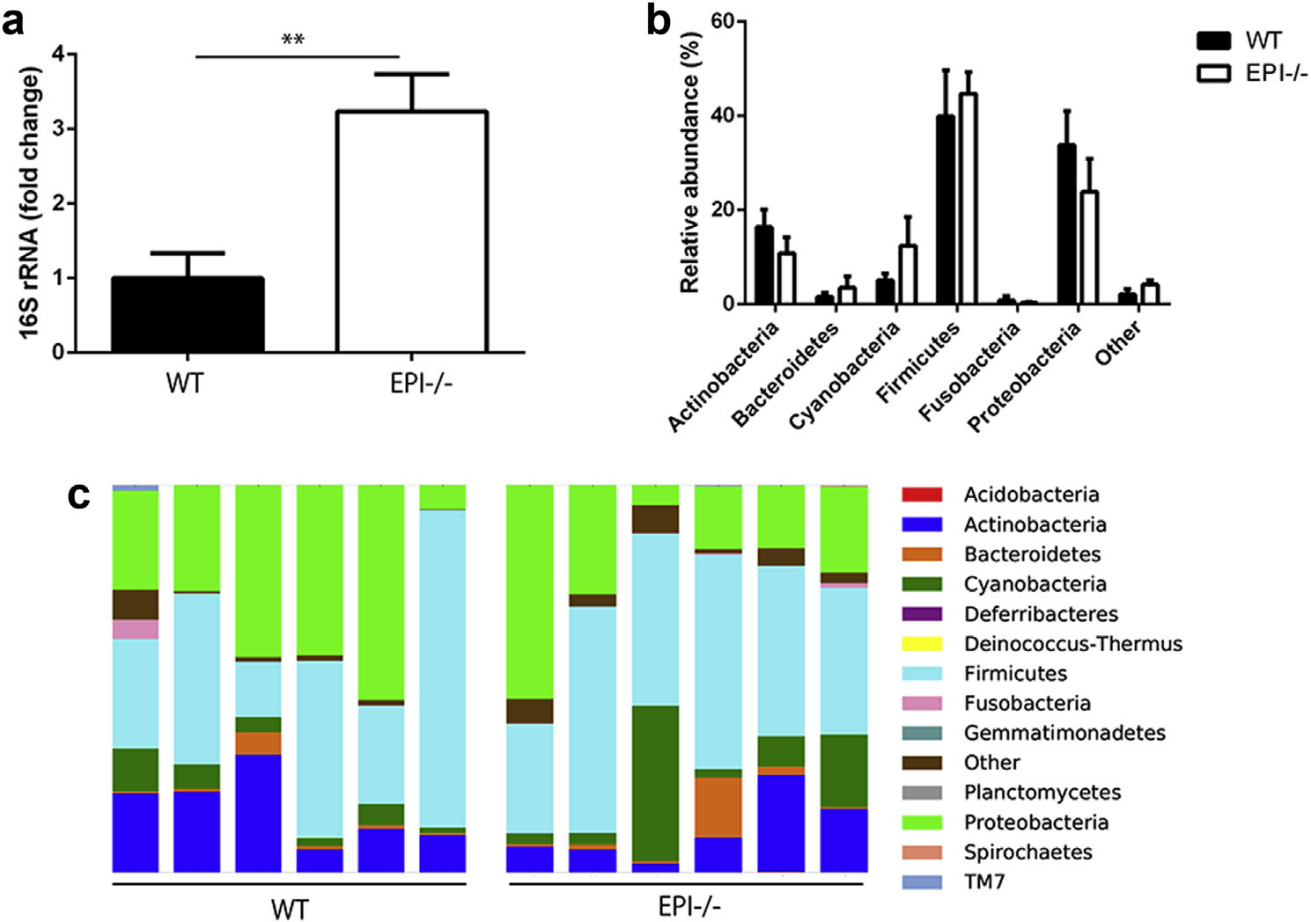
**Characterization of skin microbiota in EPI-/- and WT mice.** (**a**) qPCR of 16sRNA gene in EPI-/- and WT ear skin. Data are means ± SEM from six mice per group. **P < 0.01. (**b** and **c**) Microbial diversity of EPI-/- and WT ear skin obtained by 16s RNA gene pyrosequencing from six mice per group. (**b**) Data are presented as means of relative abundance ± SEM. (**c**) Each column shows relative abundance of the bacterial phyla in a single mouse. There were no statistically significant differences between WT and EPI-/- skin microbiota phyla.

To determine whether EPI-/- mice had an altered skin microbiome, we measured the quantity and diversity of microbes on the ear skin of six EPI-/- and six WT control mice on the same genetic background. Quantitative PCR (qPCR) of the 16S rRNA gene revealed that the bacterial load of EPI-/- mice skin was significantly higher than that of control mice (Figure 1a). However, pyrosequencing of 16S rRNA gene PCR products showed that the composition of the bacterial flora of EPI-/- and control skin did not differ significantly (Figure 1b and c).

**Figure 2:**
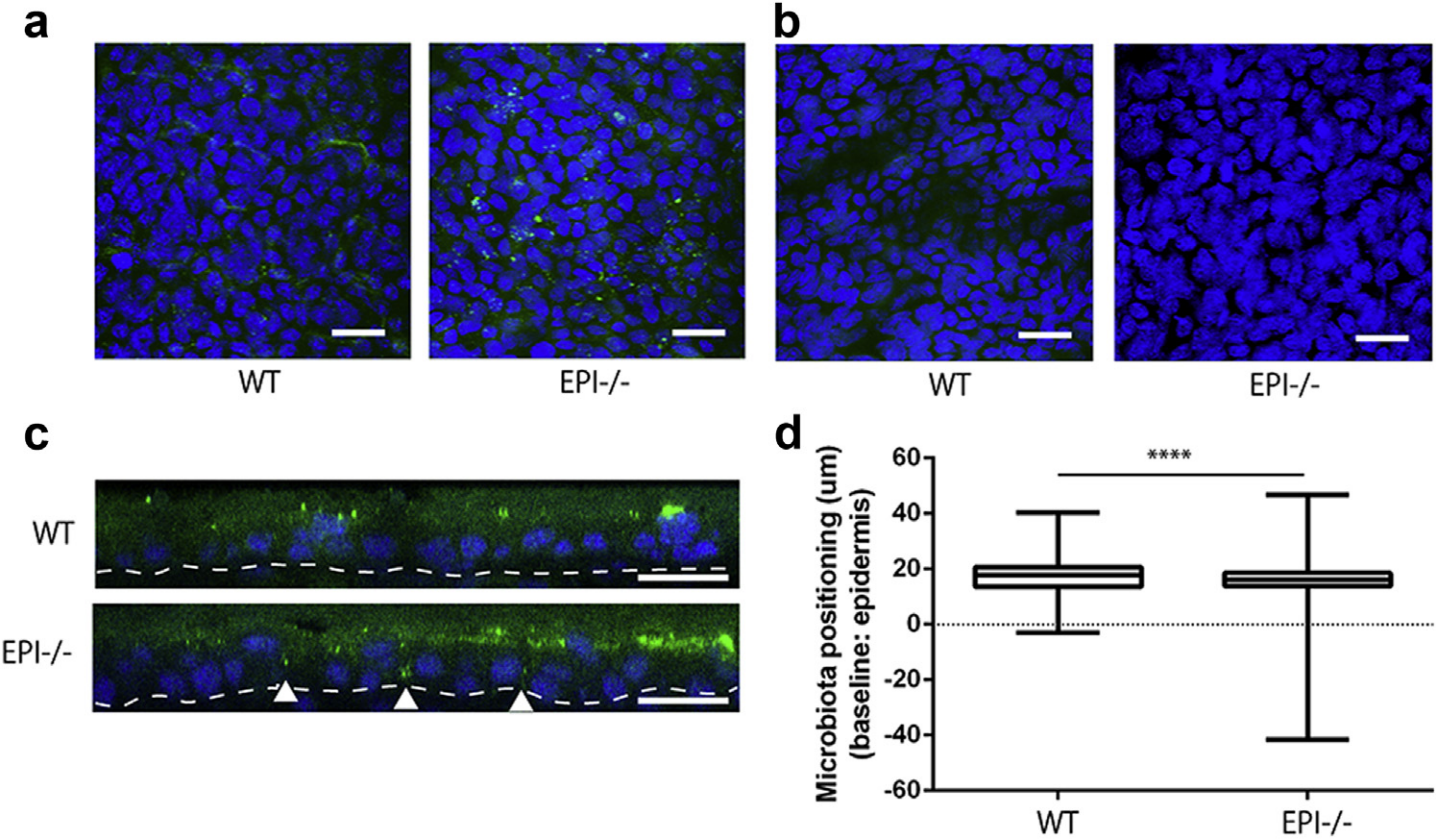
**Epidermal penetration by bacteria.** (**a** and **b**) Whole-mount FISH of ear skin using BacUni (**a**) or *C. dubuliniensis* (**b**) probes (green), with DAPI counterstain (blue). The images are Z-stack maximum projections of the epidermis. Scale bars = 20 μm. (**c**) Optical sectioning of 3D reconstructed whole-mount FISH (BacUni). Arrowheads show bacterial penetration of the basal epidermal layer. Dotted line indicates position of basement membrane. Scale bars = 10 μm. (**d**) Quantitation of location of individual BacUni signals relative to the median thickness (dotted line) of the epidermis. The boxes extend from the 25th to 75th percentiles. Whiskers indicate minimum to maximum. ****P < 0.0001.

The microbiota of EPI-/- skin were visualized by whole-mount fluorescence in situ hybridization (FISH) of the epidermis with a universal bacterial probe. There was punctate labeling on the surface of both EPI-/- and control skin (Figure 2a). A FISH probe for *Candida dubulinienesis* served as a negative control (Biasoli et al., 2010) (Figure 2b). In WT epidermis, bacteria were largely confined to the cornified layers, which can be distinguished in vertical sections because the cells lack nuclei (Figure 2c). However, bacteria in EPI-/- epidermis were also localized between keratinocytes in the living layers, extending in some cases into the basal layer (arrowheads, Figure 2c). Quantitation of the label confirmed that bacteria in EPI-/- epidermis were located significantly deeper than in control epidermis (Figure 2d; WT vs. EPI-/- : 17.13 ± 0.04 vs. 15.89 ± 0.02 μm to the median line of epidermis).

### Atopic features of EPI-/- mouse skin are unaffected by skin microbiota

**Figure 3:**
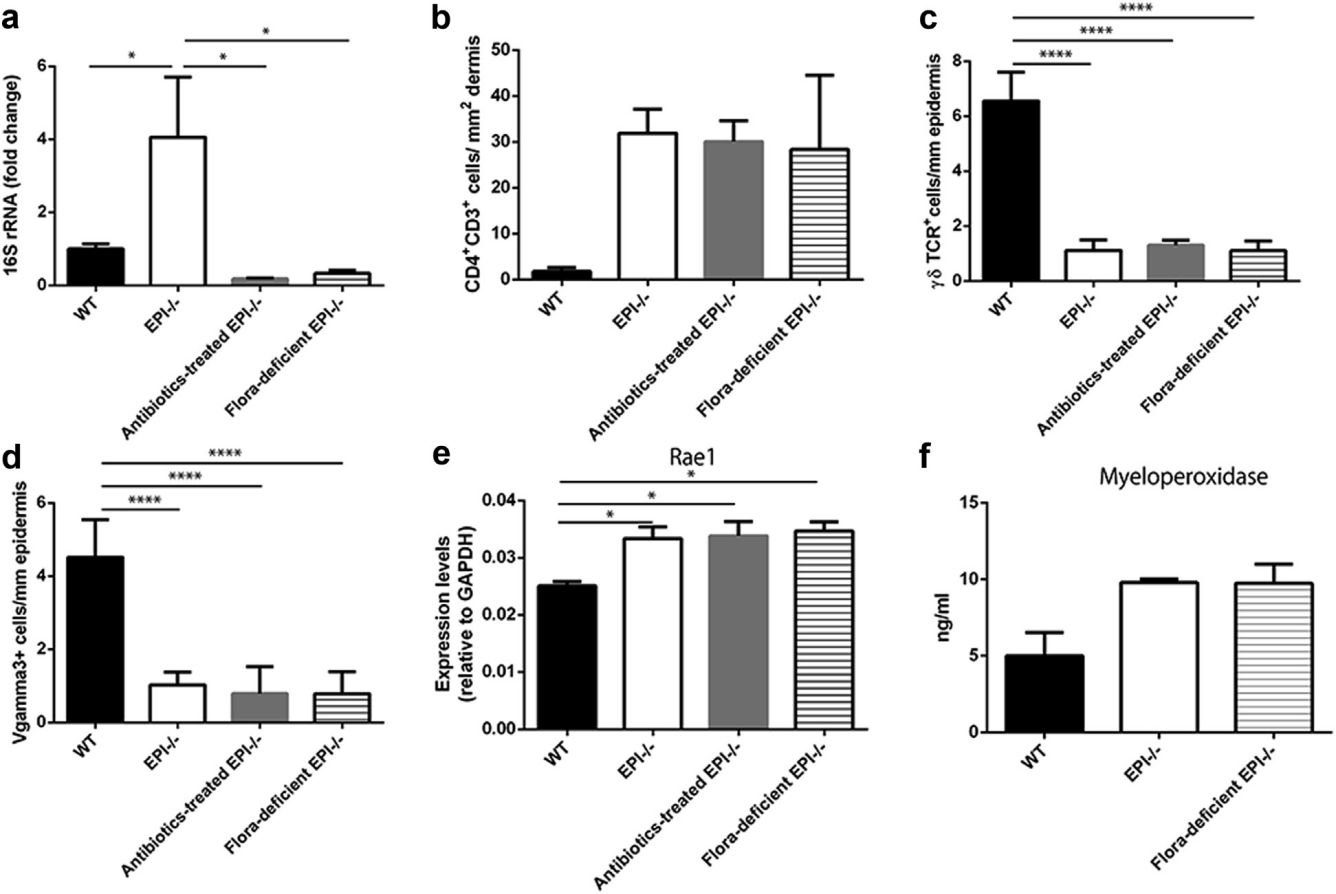
**Microbiota independent inflammatory and stress response phenotypes of EPI-/- skin.** (**a**) qPCR of 16s RNA gene in WT, EPI-/-, antibiotic-treated EPI-/-, and flora-deficient EPI-/- ear skin samples. (**b**) Number of CD4^+^CD3^+^ cells per mm^2^ dermis. (**c**) Number of γδTCR^+^ cells per mm epidermis. (**d**) Number of Vγ3^+^ cells per mm epidermis. (**e**) qRT-PCR of *Rae-1* in WT, EPI-/-, antibiotic-treated EPI-/-, and flora-deficient EPI-/- epidermis. (**f**) Quantification of MPO in WT, EPI-/-, and flora-deficient skin lysates. Data are means ± SEM from at least four (**a**) or three (**b–f**) mice per group. *P < 0.05, ****P < 0.001; unless indicated no significant differences were found (one-way ANOVA followed by Tukey’s test).

To determine whether the increased bacterial load influenced the atopic phenotype of EPI-/- mice, we used two strategies to reduce the bacterial content of the skin. One was to breed EPI-/- mice for multiple generations under specific-pathogen-free (SPF) barrier conditions (flora-deficient EPI-/- mice). The other was to treat them systemically for 2 weeks with the broad-spectrum antibiotic enrofloxacin, a time frame chosen on the basis that the exaggerated atopic response to TPA we described previously (Cipolat et al., 2014) is induced within 9 days. Both treatments reduced the bacterial load of EPI-/- skin to that of WT control mice (Figure 3a).

As previously reported (Cipolat et al., 2014, Sevilla et al., 2007), EPI-/- mice have more dermal CD4^+^CD3^+^ lymphocytes than control mice (Figure 3b). This difference persisted when EPI-/- mice were depleted of bacteria (Figure 3b). The number of epidermal γδTCR^+^CD3^+^ lymphocytes (dendritic epidermal T cells) is reduced in EPI-/- mice (Cipolat et al., 2014, Sevilla et al., 2007), and again this was not affected by reducing the number of skin bacteria, when evaluated by expression of a pan-γδTCR marker (Figure 3c) or Vγ3, a specific marker of epidermal dendritic epidermal T cells (Figure 3d).

Dendritic epidermal T cells are involved in the lymphoid stress-surveillance response, in which tissue damage upregulates “stress-associated” genes, including *Rae-1*, in keratinocytes, leading to the activation of immunoreceptor NKG2D of immune cells (Strid et al., 2009). *Rae-1* expression was increased in EPI-/- mice, as described previously (Cipolat et al., 2014), and was not reduced by depletion of the skin microbiota (Figure 3e).

Neutrophil infiltration, indicative of chronic inflammation, is highly elevated in TPA-treated EPI-/- skin (Cipolat et al., 2014). There was a slight (but not statistically significant) increase in the level of myeloperoxidase (MPO), a marker of neutrophil activation, in untreated EPI-/- skin (Figure 3f). MPO activity was not, however, affected by depletion of the skin microbiota (Figure 3f). These findings were supported by quantitating the number of Ly6G/Gr-1 positive cells (neutrophils) in the dermis of WT, EPI-/-, flora-deficient EPI-/-, and antibiotic-treated EPI-/- mice (see Supplementary Figure 1, online).

**Figure 4:**
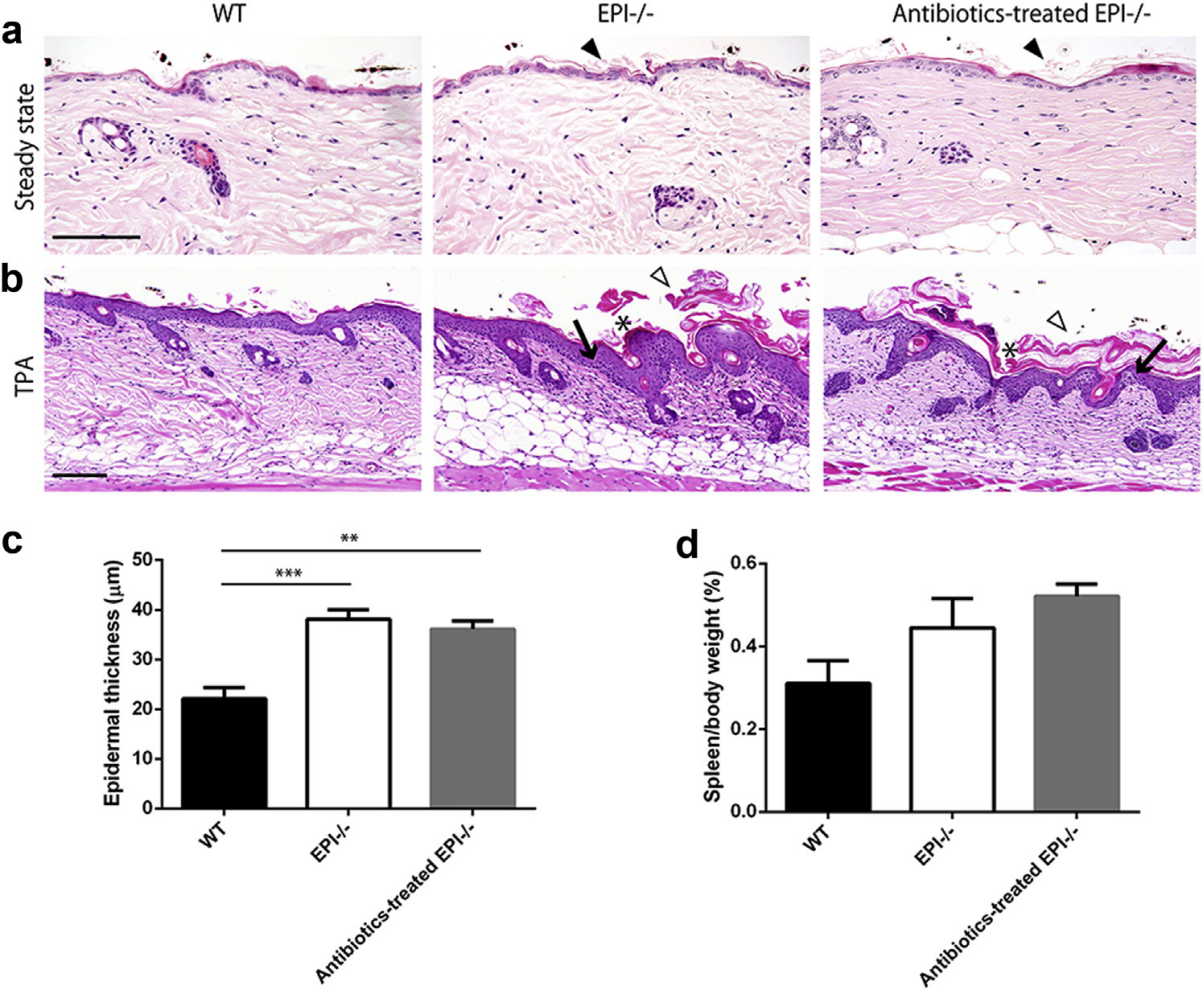
**Effect of antibiotic treatment on skin histology.** (**a**) Hematoxylin and eosin-stained skin sections of WT, EPI-/-, and antibiotic-treated EPI-/- mice at steady state. Arrowheads, mild hyperkeratosis. (**b**) Hematoxylin and eosin-stained sections of WT, EPI-/-, and antibiotic-treated EPI-/- skin after three applications of TPA. White arrowheads, severe hyperkeratosis; asterisks, parakeratosis; black arrows, spongiosis. Scale bars = 100 μm. (**c** and **d**) Epidermal thickness (**c**) and spleen mass (**d**) of WT, EPI-/-, and antibiotic-treated EPI-/- mice. TPA was applied three times on alternating days on all mice (n = 3 or 4 per condition). **P < 0.01, ***P < 0.001.

Histologically, untreated adult dorsal skin of EPI-/- mice showed mild hyperkeratosis (Figure 4a) and an exaggerated epidermal response to TPA that included hyperkeratosis, parakeratosis, epidermal thickening, and spongiosis (Figure 4b and c), as reported previously (Cipolat et al., 2014, Sevilla et al., 2007). Depletion of the skin microbiota by antibiotic treatment had no effect on these features (Figure 4a–c). In addition, antibiotics did not reduce spleen size in EPI-/- mice (Figure 4d).

### EPI-/- mice have an increased level of epidermal AMPs, proteases, and protease inhibitors, independent of skin microbiota

**Figure 5:**
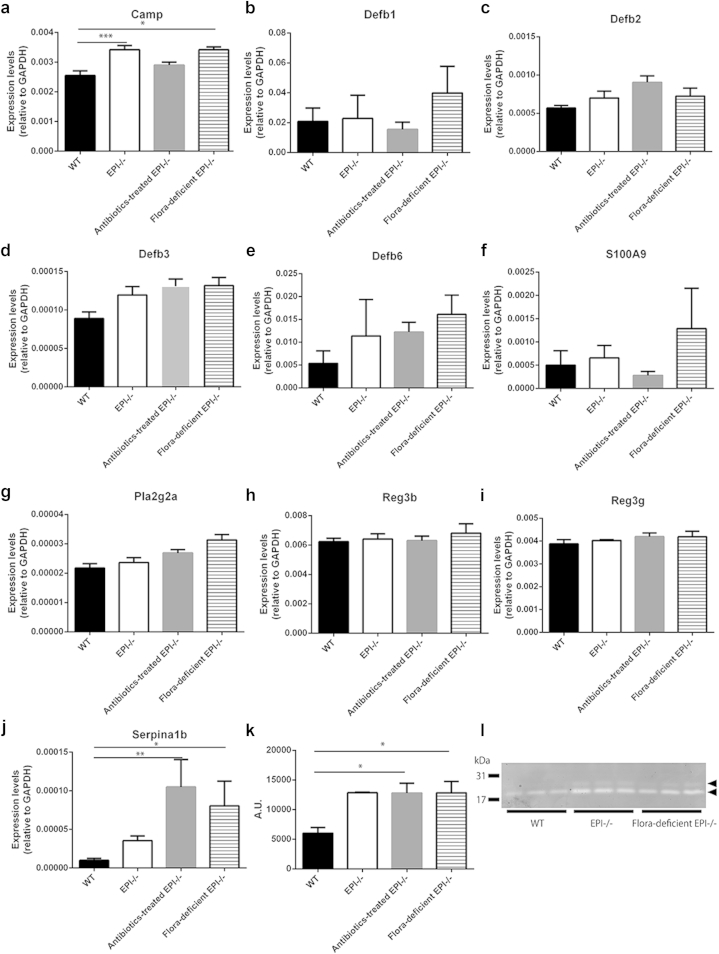
**AMP expression and protease activity in EPI-/- epidermis after microbiota modulation.** (**a–k**) qRT-PCR of *Camp* (**a**) , *Defb1* (**b**), *Defb2* (**c**), *Defb3* (**d**), *Defb6* (**e**), *S100A9* (**f**), *Pla2g2a* (**g**), *Reg3b* (**h**), *Reg3g* (**i**), and *Serpina1b* (**j**) in WT, EPI-/-, antibiotic-treated EPI-/-, and flora-deficient EPI-/- epidermis. Data are means ± SEM from at least four mice per group. *P < 0.05, **P < 0.01, ***P < 0.001. (**k**) Total epidermal protease activities of WT, EPI-/-, antibiotic-treated EPI-/-, and flora-deficient EPI-/- skin. Data are means ± SEM from three to four mice per group. (**l**) Casein gel electrophoresis of epidermal lysates. Each lane is lysate from a different mouse. Positions of molecular mass markers (31 kDa, 17 kDa) are shown. The two major bands of proteolytic activity are indicated by arrowheads.

Because bacterial load was not responsible for the skin inflammatory phenotype or keratinocyte-specific stress response of EPI-/- mice, we next investigated whether the expression of AMPs was influenced by skin bacteria. Skin AMPs belong to several distinct protein families that target different types of bacteria (Gallo and Hooper, 2012). We examined expression of representatives of three of the families by qPCR. EPI-/- epidermis had significantly elevated levels of *Camp* (cathelicidin-related antimicrobial peptide; gram-positive and -negative bacteria). *Defb1*, *Defb2*, *Defb3*, and *Defb6* (β-defensins; gram-positive and -negative bacteria) and *S100A9* (calprotectins; *Staphylococcus aureus*) levels were similar to control mice (Figure 5a–f). Expression of AMP genes was not affected by depletion of the skin microbiota (Figure 5a–f). Immunofluorescence labeling for β3-defensin and *Camp* in sections of mouse back skin suggested that both EPI-/- and flora-deficient EPI-/- epidermis had more abundant β3-defensin and *Camp* than WT epidermis (see Supplementary Figure 2, online).

Antibiotic treatment reduces expression of intestinal AMPs, including *Pla2g2a*, *Reg3b*, and *Reg3g* (Reikvam et al., 2011). Intriguingly, these genes were not upregulated in EPI-/- skin and were not affected by the depletion of skin microbiota (Figure 5g–i). This suggests that the AMPs of the gut and skin respond differently to bacterial load.

Epidermal proteases are known to process native AMPs to their active forms (Ovaere et al., 2009), and expression of one of the major skin serine protease inhibitors, *Serpina1b*, is increased in EPI-/- skin (Sevilla et al., 2007). In contrast to AMPs, *Serpina1b* levels were affected by depletion of skin bacteria in EPI-/- mice, showing a significant increase in antibiotic-treated and SPF-housed animals (Figure 5j). However, when WT mice were treated with antibiotics, epidermal *Serpina1b* expression was not altered (see Supplementary Figure 3, online).

In contrast to our earlier report (Sevilla et al., 2007), total epidermal protease activity was increased in EPI-/- mice. However, total protease activity was not affected by bacterial depletion (Figure 5k). Casein gel electrophoresis revealed two predominant proteins with protease activity, which, based on electrophoretic mobility, likely correspond to elastase 2 and kallikrein 7 (Figure 5l). Both bands were more abundant in EPI-/- than WT skin but were not reduced by bacterial depletion (Figure 5l). These data indicate that, like AMP expression, total proteases are unaffected by bacterial load in the skin of EPI-/- mice, notwithstanding the increase in *Serpina1b* levels.

## Discussion

Our study demonstrates that the epidermal barrier defect of EPI-/- mice results in an increased bacterial load and deeper bacterial penetration of the epidermis. Both phenomena are most likely attributable to the previously reported fragility of the cornified envelope and abnormalities in the intercellular desmosomal junctions (Sevilla et al., 2007). The increase in bacteria does not, however, impact on the atopic phenotype of EPI-/- skin, including inflammation, upregulation of *Rae-1*, AMPs, and epidermal protease activity, and does not reflect any major changes in skin bacterial phyla.

The only exception to these observations was *Serpina1b*, which, as reported previously (Sevilla et al., 2007), was elevated in EPI-/- skin relative to WT. *Serpina1b* levels were further increased by bacterial depletion in EPI-/- but not WT skin. Although the underlying mechanism and biological significance of the *Serpina1b* findings remain to be explored, it is worth noting that elevated expression of Serpins contributes to barrier dysfunction and inflammation in allergen-treated mouse skin (Sivaprasad et al., 2015). It would be interesting to examine whether EPI-/- mice exhibit increased allergic contact dermatitis, because Filaggrin-null mice, which also have a defective barrier, display an enhanced cutaneous response to ovalbumin sensitization (Kawasaki et al., 2012).

Our observations indicate that the major tumor-protective mechanism identified in EPI-/- mice, namely signaling via Rae-1 and NKG2D, is attributable to the inherent structural defects in the epidermal barrier and not to the increase in the skin microbiota. This is in marked contrast to the protumorigenic effect of flagellated bacteria that colonize skin wounds and signal via Toll-like receptor-5 (Hoste et al., 2015a, Hoste et al., 2015).

Our study distinguishes phenotypes resulting from changes to the epidermal barrier from microbiota-induced phenotypes. Genetic ablation of the serine protease matripase, which processes Filaggrin and is required for normal cornified envelope assembly, results in changes in the composition of the skin microbiota and changes in AMP levels, but whether the two phenomena are related has not been explored (Scharschmidt et al., 2009). The AMP β-defensin-2 is upregulated in the skin of patients with atopic dermatitis (Asano et al., 2008, Ong et al., 2002), and *S. aureus* infections are common in these patients (Ogawa et al., 1994). However, the skin inflammation that is a hallmark of atopic dermatitis and other epidermal barrier disorders, such as congenital ichthyosis (Kabashima, 2013, Oji et al., 2010), is not alleviated by antibiotic treatment. We propose that the altered skin microbiome associated with atopic dermatitis is a consequence, not a cause, of the disease.

## Methods

### Mice

EPI-/- mice were generated as described previously on a mixed genetic background (Sevilla et al., 2007). Genome scanning performed by the Jackson Laboratory (Bar Harbor, ME; Cipolat et al., 2014) established that their genetic background comprises Sv129 (40.98% ± 1.52%), C57Bl/6 (51.39% ± 1.62%), and BALB/c (4.7% ± 1.24%). On that basis we selected the F2 generation of Sv129 and C57BL/6J crosses (51.82% ± 3.35% Sv129; 49.92% ± 1.98% C57Bl/6) as the control for all experiments, because their genetic background was 95% identical to that of EPI-/- mice. The F2 control mice were previously used in studies of the susceptibility of EPI-/- mice to chemical carcinogenesis (Cipolat et al., 2014). Matched control and EPI-/- mice were housed in the same environments and had the same diet.

For some experiments multiple generations of EPI-/- and control mice were housed under SPF conditions. Analysis was performed on mice that had been born in pathogen free conditions in an SPF barrier facility. Entry into the facility required staff to change into scrubs and protective footwear, to take an air shower, and to use hand disinfectant. Mice, whether behind the barrier or in the conventional facility, were kept in individually ventilated cages that were only opened in a laminar flow cabinet and were only handled with gloved hands. For antibiotic treatment oral enrofloxacin (5 mg/kg/day) was given to mice kept in the conventional facility for 2 weeks.

For TPA treatment, 6 nmol (3.7 μg) of TPA (Sigma-Aldrich, Gillingham, UK) in 200 μl acetone was applied to mouse dorsal skin three times on alternating days (Cipolat et al., 2014). Skin, and in some cases spleen, was harvested 48 hours after the last TPA treatment. Epidermal thickness was assessed on hematoxylin and eosin-stained sections of TPA-treated skin using ImageJ software (NIH, Bethesda, MD). At least six fields were analyzed per skin section. All experiments were performed under the terms of a UK government Home Office project license following institutional ethical review.

### Analysis of bacterial load

Extraction of skin bacterial DNA was performed as described previously (Scharschmidt et al., 2009). Briefly, 5-mm diameter skin biopsies were collected from the ears of age- and sex-matched mice using sterile instruments. DNA was extracted with the PureLink Genome DNA Mini Kit (Life Technologies, Grand Island, NY) following the bead-beating step using Precellys 24 (Bertin Technologies, Montigny le Bretonneux, France). 16S rRNA gene qPCR was carried out using specific primers (Bact-8F: AGAGTTTGATCCTGGCTCAG, Bact–338R: CTGCTGCCTCCCGTAGGAGT) with fast SYBR Green detection (Applied Biosystems, Foster City, CA) and run on an ABI Prism 7900HT Sequence detection system (Applied Biosystems). Standard curves were prepared by amplifying serial dilutions of known quantities of *E. coli* cells. The V1-V2 region of the 16S rRNA gene was amplified using barcoded fusion primers, and PCR products were sequenced on a 454GS FLSGX platform (Roche, Branford, CT). Pyrosequencing reads were uploaded into QIIME (qiime.org/) and processed as described previously (Caporaso et al., 2010).

### Whole-mount FISH

Pieces of ear skin were collected with a sterilized 3-mm biopsy punch. Skin samples were fixed in 1:1 acetone-to-methanol and incubated with PNA FISH probes at 55 °C. A universal bacteria probe (BacUni; AdvanDx, Woburn, MA) and probe for *C. dubuliniensis* (AdvanDx) were used. DAPI was used for nuclear staining. Images and Z-stacks of whole-mount FISH were obtained using a TCS SP5 Tandem Scanner confocal microscope (Leica, Mannheim, Germany). Optical sectioning and Z-stack maximum projection images of whole-mount preparations were produced using LAS AF software (Leica). Evaluation of bacterial location was performed on three-dimensional reconstructed skin using Volocity software (PerkinElmer, Wellesley, MA). All the BacUni DAPI signals were detected using the software. The median epidermal line was calculated using the localization of the center of epidermal DAPI signals. The distance of each BacUni signal from the median epidermal line was plotted. Positive values indicate signals above the median line, whereas negative values correspond to signals below the median line.

### Immunofluorescence

Frozen sections were fixed with 4% paraformaldehyde/phosphate-buffered saline and stained with the following antibodies conjugated with Alexa 488 or FITC or Alexa 633: anti-CD3 (BD Pharmingen, San Diego, CA, clone 17A2), anti-CD4 (eBioscience, San Diego, CA, clone RM4-5), anti-γδ TCR (BD Pharmingen, clone GL3), anti-Vγ3 TCR (BD Pharmingen, clone 536), or anti-Ly6G/Gr-1 (Beckman Coulter, Miami, FL, clone RB6-8C5). Formalin-fixed paraffin sections were treated with citrate buffer for antigen retrieval and incubated with anti-Camp (Abcam, Cambridge, UK) or anti-β3 defensin (Alpha Diagnostic, San Antonio, TX) followed by Alexa 488-conjugated donkey anti-rabbit antibody (Life Technologies). DAPI or PI was used for nuclear staining. Images were acquired using a TCS SP5 Tandem Scanner or an FV1000 confocal microscope (Olympus, Tokyo, Japan). When quantifying cells that expressed a particular marker, at least six fields were analyzed per skin section.

### MPO ELISA

For MPO quantification, an MPO ELISA kit (Hycult Biotech, Uden, Netherlands) was used according to the manufacturer’s protocol. A 3-mm punched ear skin punch biopsy was homogenized in lysis buffer (200 mM NaCl, 5 mM EDTA, 10 mM Tris, 10% glycerin, 1 mM phenylmethylsulfonyl fluoride) and centrifuged. The supernatants were used for MPO ELISA. The absorbance was measured using a Sunrise spectrophotometer (Tecan, Männedorf, Switzerland).

### Quantitative reverse transcriptase PCR

RNA was extracted from murine whole skin or from epidermis that had been scraped from the skin after incubation in phosphate-buffered saline for 30 seconds at 56 °C in phosphate-buffered saline. The RNeasy mini kit (Qiagen, West Sussex, UK) was used according to the manufacturer’s instructions. RNA concentration was measured using an ND-100 NanoDrop spectrophotometer (NanoDrop Technologies, Wilmington, DE). cDNA was synthesized using a Superscript III First-Strand Synthesis Supermix for quantitative reverse transcriptase PCR (qRT-PCR) kit (Invitrogen, Carlsbad, CA) according to the manufacturer’s instructions. qRT-PCR was carried out using specific primers and fast SYBR Green and run on an ABI Prism 7900HT Sequence detection system. All samples were compared relative to mouse Gapdh as a housekeeping control.

The following primers were used. *Rae-1*: CTAGTGCCACCTGGGAATTCA (forward), CATCATTAGCTGATCTCCAGCTCA (reverse); *Camp*: CTTCAACCAGCAGTCCCTAGACA (forward), TCCAGGTCCAGGAGACGGTA (reverse); *Defb2*: GCCATGAGACTCTCTGCTC (forward), TGCAACAGGGGTTCTTCTCT (reverse); *Defb3*: ATTTCTCCTGGTGCTCGTGT (forward), GGAACTCCACAACTGCCAAT (reverse); *Pla2g2a*: CTGTTGCTACAAGAGCCTGG (forward), TTTTCTTGTTCCGGGCGAAA (reverse); *Reg3b*: CCTTAGACCGTGCTTTCTGTG (forward), GTCCATGATGCTCTTCAAGACA (reverse); *Reg3g*: ACATCAACTGGGAGACGAATC (forward), TTTGGGATCTTGCTTGTGGCTA (reverse); *Serpina1b*: CATAGGAACGGCTTCAAAGA (forward), TCCAGATCCATATCCCCAGA (reverse); *Gapdh*: AACATCAAATGGGGTGAGGCC (forward), GTTGTCATGGATGACCTTGGC (reverse).

### Epidermal protease activity

Epidermal lysates were obtained as described previously (Descargues et al., 2005). Briefly, epidermis was homogenized in 1 M acetic acid using Precellys 24 (Bertin Technologies). After overnight extraction at 4 °C, soluble proteins were lyophilized and resuspended in MiliQ water (Millipore, Bedford, MA). After acetone precipitation the BCA protein assay kit (Pierce, Rockford, IL) was used to quantify protein concentration. The soluble fraction, 2 μg, was monitored for protease activity using the EnzChek Protease Assay Kit (Life Technologies), according to the manufacturer’s instructions; 5 μg of the soluble fraction was loaded onto Novex Zymogram (casein) Gels (Life Technologies) for electrophoresis. Gels were stained with Coomassie brilliant blue.

### Statistics

Statistical analysis was performed using GraphPad Prism (GraphPad Software, San Diego, CA). Probability values were determined with the unpaired Student’s *t*-test or one-way ANOVA followed by Tukey’s test. For analysis of bacterial depth in the skin, a nonparametric Mann-Whitney test was performed.

## Conflict of Interest

The authors state no conflict of interest.
