# Corrigendum to ‘Increased Bacterial Load and Expression of Antimicrobial Peptides in Skin of Barrier-Deficient Mice with Reduced Cancer Susceptibility’ Journal of Investigative Dermatology, Volume 136, Issue 1, January 2016, Pages 99-106

**DOI:** 10.1016/j.jid.2024.07.001

**Published:** 2025-02

**Authors:** Ken Natsuga, Sara Cipolat, Fiona M. Watt

**Affiliations:** 1 Cancer Research UK Cambridge Research Institute, Li Ka Shing Centre, Cambridge, United Kingdom; 2 Department of Dermatology, Hokkaido University Graduate School of Medicine, Sapporo, Japan; 3 Centre for Stem Cells and Regenerative Medicine, King’s College London, London, United Kingdom

The authors have become aware that the WT and EPI-/- panels of Figure 4b in this publication unintentionally duplicate images published by the same authors in another journal (Cipolat S, Hoste E, Natsuga K, Quist SR, Watt FM. Epidermal barrier defects link atopic dermatitis with altered skin cancer susceptibility. eLife 5:3e01888, 2014 (https://doi.org/10.7554/elife.01888)). One of the authors of the earlier paper inadvertently sent the wrong images to the first author of the JID paper. Since the epidermal phenotype induced by TPA was the same whether or not the mice had been injected with IgG, none of the authors spotted the duplication. The corrected image appears below.

**Figure 4:**
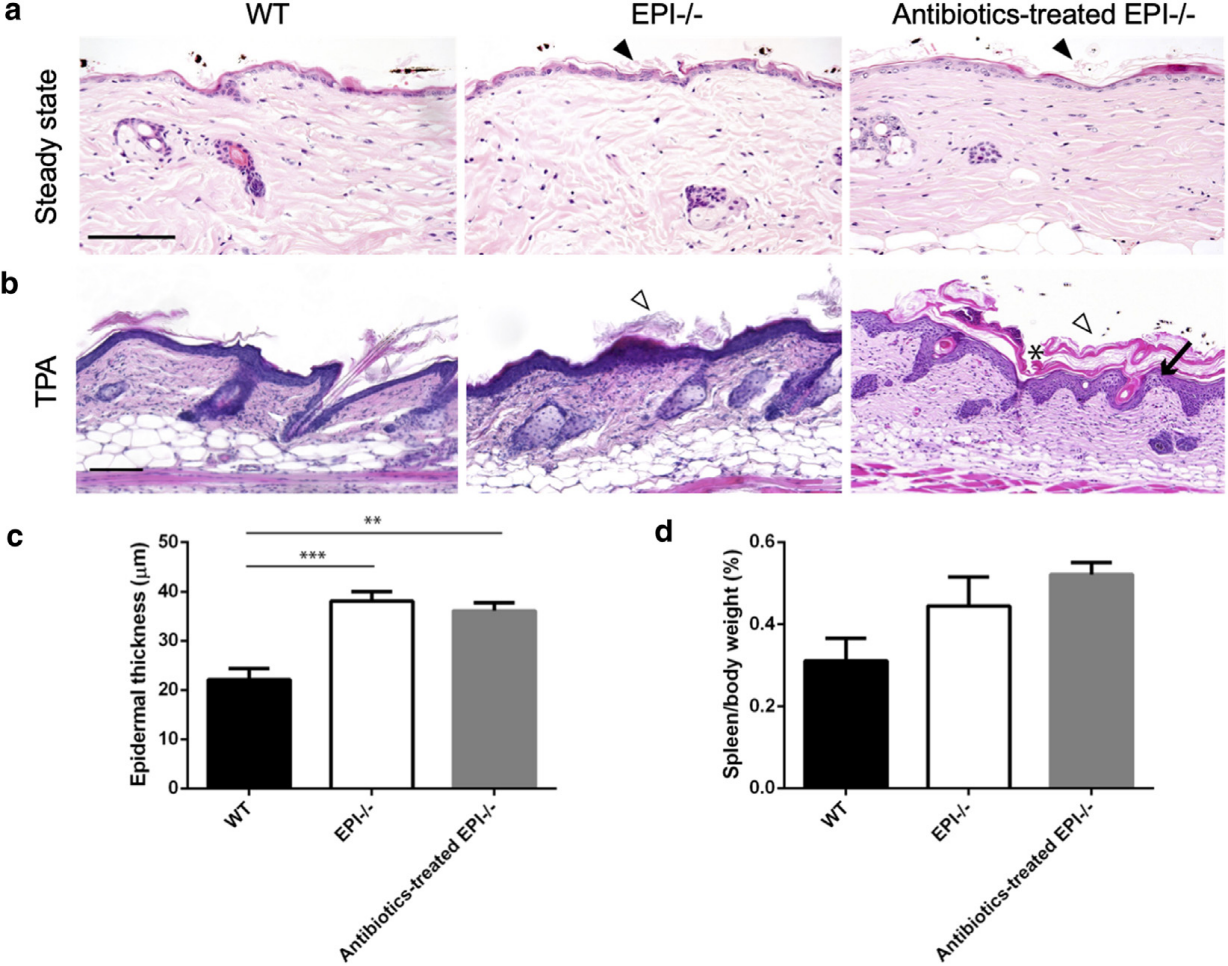
**Effect of antibiotic treatment on skin histology.** (a) Hematoxylin and eosin-stained skin sections of WT, EPI-/-, and antibiotic-treated EPI-/- mice at steady state. Arrowheads, mild hyperkeratosis. (b) Hematoxylin and eosin-stained sections of WT, EPI-/-, and antibiotic-treated EPI-/- skin after three applications of TPA. White arrowheads, severe hyperkeratosis; asterisks, parakeratosis; black arrows, spongiosis. Scale bars = 100 μm. (c and d) Epidermal thickness (c) and spleen mass (d) of WT, EPI-/-, and antibiotic-treated EPI-/- mice. TPA was applied three times on alternating days on all mice (n = 3 or 4 per condition). ∗∗P < 0.01, ∗∗∗P < 0. 001.

The authors would like to apologize for any inconvenience caused.

