# Supplementary material for: Increased Bacterial Load and Expression of Antimicrobial Peptides in Skin of Barrier-Deficient Mice with Reduced Cancer Susceptibility
Source: J Invest Dermatol. 2016 Jan;136(1):99–106. doi: 10.1038/jid.2015.383 (PMC4759621; doi:10.1038/jid.2015.383)
Supplement: Supplementary Figures 1–3 [file mmc1.pdf]

Supplementary Figures

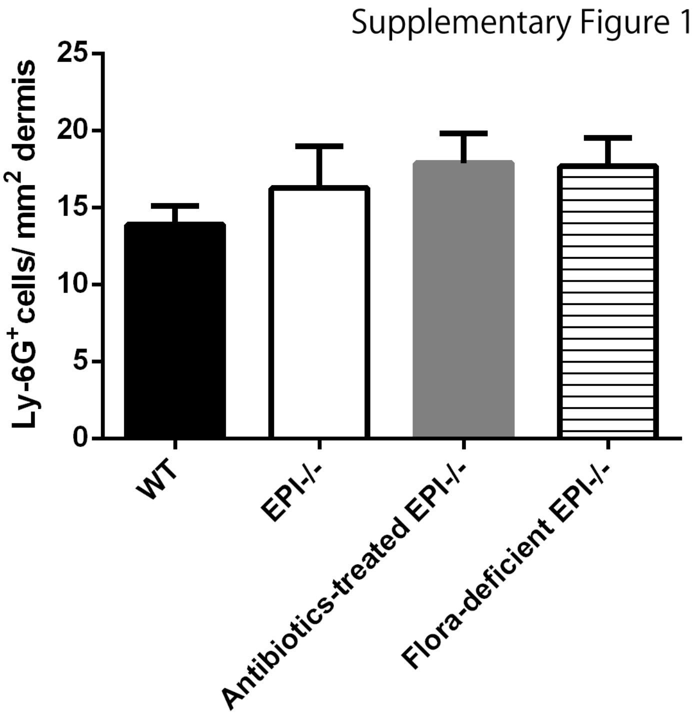

Supplementary Figure 1. Quantification of Ly6G+ dermal cells. Number of Ly6G+ cells per mm^2 dermis in WT, EPI-/-, antibiotics-treated EPI-/- and flora-deficient EPI-/- skin. Data are means ± SEM from at least 4 mice per group. No statistically differences were found between groups.

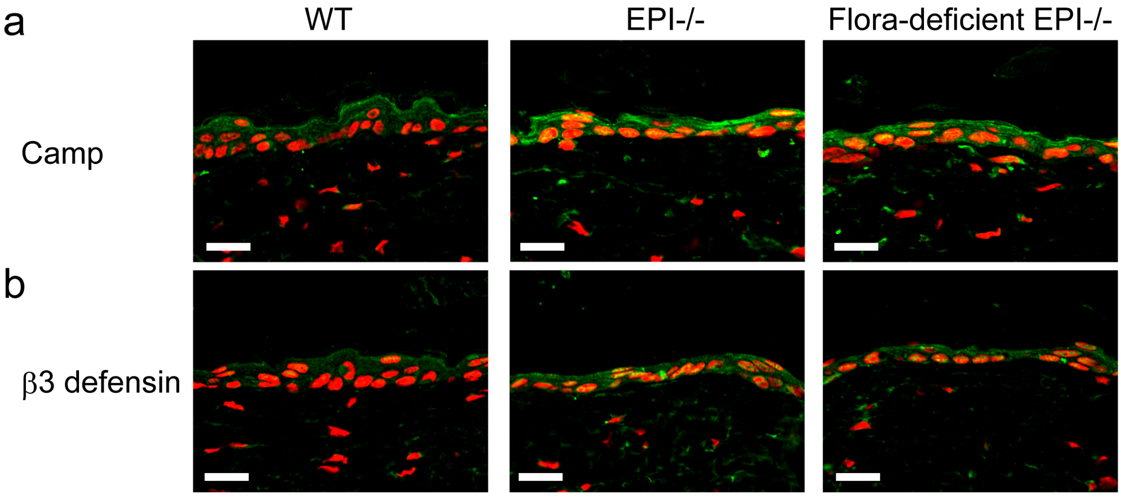

Supplementary Figure 2. AMP expression in EPI-/- epidermis. (A, B) Immunofluorescence labelling of Camp (**A**) and β3 defensin, encoded by Defb3, (**B**) in WT, EPI-/- and flora deficient EPI-/- skin (green fluorescence, with red propidium iodide nuclear counter-stain). Scale bars: 20 μm.

Supplementary Figure 3

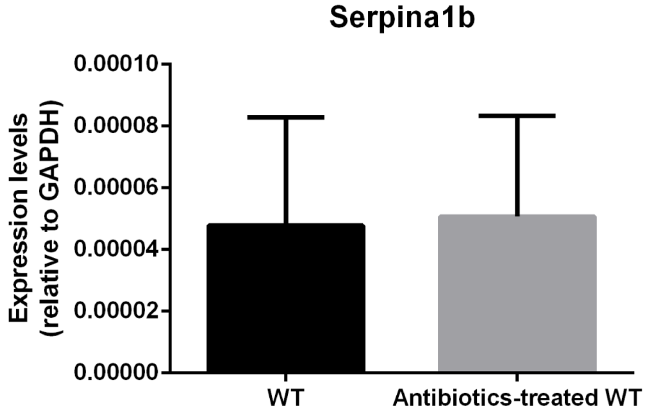

Supplementary Figure 3. Serpina1b expression in WT epidermis treated with antibiotics. qRT-PCR of Serpina1b in WT and antibiotic-treated WT epidermis. Data are means ± SEM from 4 mice per group. No statistically significant differences were found between groups.
